# Sexual Differences in Physiological and Transcriptional Responses to Salinity Stress of *Salix linearistipularis*

**DOI:** 10.3389/fpls.2020.517962

**Published:** 2020-10-19

**Authors:** Shuang Feng, Hongwei Sun, Hongping Ma, Xin Zhang, Shurong Ma, Kun Qiao, Aimin Zhou, Yuanyuan Bu, Shenkui Liu

**Affiliations:** ^1^Key Laboratory of Saline-Alkali Vegetation Ecology Restoration, Ministry of Education, Northeast Forestry University, Harbin, China; ^2^College of Horticulture and Landscape Architecture, Northeast Agricultural University, Harbin, China; ^3^The State Key Laboratory of Subtropical Silviculture, Zhejiang Agriculture and Forestry University, Hangzhou, China

**Keywords:** dioecious, *Salix linearistipularis*, salinity tolerance, sexual differences, salt-responsive genes

## Abstract

Willow (*Salix*), a dioecious plant, is an important ornamental tree species in the world. *Salix linearistipularis*, a perennial woody plant species naturally distributed on the Songnen Plain saline-alkali land in northeast China, has a high saline condition. To study the sexual differences of *S. linearistipularis* in salinity tolerance, the physiological and transcriptional responses to salinity were compared between female and male cuttings. Under salinity stress, the female leaves exhibited higher superoxide dismutase and peroxidase activities and photosynthetic capacity, and lower H_2_O_2_ contents than those of male leaves. Under salinity stress, sodium (Na^+^) accumulation in female leaves was lower than that in the male leaves. The non-invasive micro-test showed that the net Na^+^ efflux in the salt-treated female roots was higher than that in male roots. Physiological responses revealed that female cuttings were more tolerant than males, which may be mainly due to females having lower leaf Na^+^ accumulation and higher root Na^+^ efflux capacity than males. Transcriptional analyses showed that 108 differentially expressed salt-responsive genes were identified in both female and male roots; most of these showed sexual differences in expression patterns under salinity stress. RNA-seq combined with qPCR analysis showed that the salt-induced expression of four Na^+^/H^+^ antiporter (*NHX*) genes (*SlNHX3, 5, 6, 7*) in female roots was higher than that in male roots. Transcriptional analyses revealed that the higher Na^+^ efflux capacity in female roots than in male roots may be closely related to the differential expression of salt-responsive genes, especially *NHX* genes.

## Introduction

Soil salinity is one of the major environmental factors influencing the productivity of agriculture and forestry. To survive under adverse conditions, plants have evolved intricate salt tolerance mechanisms, especially halophytes growing in high saline soils (Deinlein et al., 2014). Generally, halophytes can exclude more sodium (Na^+^) through their roots than glycophytes under salinity stress (Garthwaite et al., 2005). Investigation of salt tolerance mechanisms could facilitate the identification of plants that better cope with salinity. Moreover, the salt tolerant plants are an important resource for the exploitation and improvement of saline soils.

Willow (*Salix*), as one of the most important landscaping greening tree species, has wide distribution and high adaptability (Zhang et al., 2017). Because of their rapid growth, high biomass yield, and ease of propagation, willows are important wood resources for both bioenergy production and afforestation (Greger and Landberg, 1999; Karp et al., 2011). Furthermore, willow species are extensively used in phytoremediation activities (Tozser et al., 2017). *Salix linearistipularis* (Franch.) Hao, a member of the Salicaceae family, is a woody plant naturally distributed in the Songnen Plain saline-alkali land in the northeast of China. *S. linearistipularis* exhibits high tolerance to salinity; therefore, it can be used for the greening and afforestation of saline-alkali land, which highlight its high and has great ecological and economic potential.

*Salix linearistipularis* is a dioecious plant. In many dioecious plant species, there are differences in morphology, physiology, and heredity, as well as environmental adaptability between female and male plants (Eckhart and Chapin, 1997; Li et al., 2007; Melnikova et al., 2017). For example, in poplars (*Populus* spp.) from the Salicaceae family, many studies have shown sexual differences between females and males in morphological, physiological, and transcriptional responses to environmental stress (Chen F. et al., 2010; Chen L. et al., 2010; Zhang et al., 2011; Jiang et al., 2012; Peng et al., 2012; Melnikova et al., 2017; Song et al., 2019). In *P. cathayana*, the growth rate, the leaf morphology changes, the dry matter and the Na^+^ accumulation, and the photosynthetic capacity in female and male cuttings under salinity stress were significantly different (Chen F. et al., 2010). Furthermore, the difference in degradation rate and abundance of proteins involved in photosynthesis, hydrogen peroxide (H_2_O_2_) scavenging, and stress response between female and male cuttings was observed under salinity stress (Chen et al., 2011). In *P. yunnanensis*, female cuttings exhibited higher sensitivity to salinity and drought than male cuttings, as well as higher sensitivity to the combination of salinity and elevated CO_2_ levels (Chen L. et al., 2010; Li et al., 2013). The transcription profiling revealed a difference in the expression of most genes involved in photosynthesis in both female and male *P. yunnanensis* under salinity stress (Jiang et al., 2012). Moreover, male *P. deltoides* cuttings exhibited competitive advantages over females under salinity stress (Li et al., 2016). Overall, these studies have shown the sexual difference in salinity tolerance between female and male plants in the genus *Populus*. The genus *Populus* and the genus *Salix* are members of Salicaceae. Unlike the male-biased *Populus*, *Salix* shows a female-biased sex ratio in its natural habitats (Dudley, 2006; Ueno et al., 2007; Hughes et al., 2010; Liao et al., 2019). A recent study has demonstrated that *Salix paraplesia* females exhibit superior drought tolerance and self−protective capacity to males at high altitudes (Liao et al., 2019). However, it remains unclear whether there are sexual differences in salinity tolerance between females and males in the genus *Salix*.

To investigate potential differences in physiological and transcriptional response of female and male *S. linearistipularis* cuttings, we measured some physiological parameters (including antioxidant enzyme activity, chlorophyll pigments, H_2_O_2_, and sodium (Na^+^) contents, and root Na^+^ efflux rate) of female and male cuttings following exposure to salinity stress. In addition, we analyzed the transcription and expression profiles of salt-responsive genes in female and male roots exposed to salinity stress. The results of the present study could enhance our understanding of sexual differences in the genus *Salix* and facilitate its effective exploitation.

## Materials and Methods

### Plant Materials and Growth Conditions

Annual female and male cuttings from *S. linearistipularis* trees (sampled in five populations) were collected from Anda Experimental Base of Northeast Forestry University of China (Anda City, Heilongjiang Province; 46°27, N, 125°22, E). The cuttings were grown in a greenhouse under a 12-h light/12-h dark photoperiod (100 μmol m^–2^ s^–1^ light) at 24°C. Female and male cuttings were planted in plastic pots containing the same homogenized soil. After 1 month, healthy plants with equivalent height and similar crown size were selected for the experiments. A female tree and a male tree from each population were also randomly selected for different treatments.

### Measurement of Antioxidant Enzyme Activities and H_2_O_2_ and Malondialdehyde Contents

The leaves of female and male plants were used to prepare leaf disks (1 cm^2^), which were immediately immersed in different concentrations of NaCl solution (0, 50, 100, 200, 300, and 400 mM) for 48 h. Water was used as the control. A total of 100 mg of the salt-treated and control samples were collected and weighed. The superoxide dismutase (SOD) and peroxidase (POD) activities, and H_2_O_2_ and MDA contents were measured using detection kits (SOD-1-Y, POD-1-Y, H_2_O_2_-1-Y, and MDA-1-Y; Comin, Suzhou, China), according to the manufacturer’s instructions.

### Measurement of Chlorophyll Content and Chl Fluorescence Parameters

Chlorophyll (Chl) was extracted from leaf samples using 80% ice-cold acetone. The absorbances of Chl a (646 nm) and Chl b (663 nm) were determined using a UV/Vis spectrophotometer. The total Chl content was the sum of Chl a and Chl b. The maximal photochemical efficiency (*F*_*v*_/*F*_*m*_), minimal fluorescence yield (*F*_0_), and maximal fluorescence yield (*F*_*m*_) were measured using an Imaging-PAM Chlorophyll Fluorometer (Walz, Germany) as described previously (Wang et al., 2014).

### Measurement of Na^+^ Contents

Female and male cuttings were exposed to 0 and 100 mM NaCl salinity solution for 12 and 48 h. After salinity treatment, the leaf samples of the cuttings were collected and dried. The dried samples were weighed and then digested in 8 mL HNO_3_ and 3 mL H_2_O_2_ for 50 min at 180°C using a microwave digestion instrument (Milestone, Italy). The Na^+^ contents in the leaves were measured by inductively coupled plasma optical emission spectrometry (ICP-OES, Perkin Elmer, United States).

### Net Na^+^ Flux Measurements

Net fluxes of Na^+^ were measured using Non-Invasive Microtest Technology (NMT) (NMT100 Series, YoungerUSA LLC, Amherst, MA, United States), as described previously (Sun et al., 2009; Zhou et al., 2018). Hydroponic female and male cuttings were exposed to 0 or 100 mM Na^+^ for 12 h, and root segments were immobilized in the measuring solution (0.1 mM KCl, 0.1 mM CaCl_2_, 0.1 mM MgCl_2_, 0.5 mM NaCl, and 0.3 mM MES, pH 5.8) to measure the Na^+^ flux. Each sample was measured continuously for 9 min and each root zone for 3 min. The Na^+^ flux rate, based on the voltages monitored between two points (0 and 30 μm), was calculated using iFluxes/imFluxes v1.0 software (YoungerUSA LLC, Amherst, MA, United States). The calibration slope for Na^+^ was 58.59 mV/decade. Six biological repeats were performed for each analysis. Ion flux was calculated based on Fick’s law of diffusion:

J=-D⁢(d⁢c/d⁢x)

where *J* represents the ion flux in the *x* direction, *dc/dx* is the ion concentration gradient, and *D* is the ion diffusion constant in a particular medium.

### RNA-Seq and Transcriptome Data Processing

Hydroponic cuttings of male and female plants were exposed to 0 or 100 mM NaCl for 6, 12, and 24 h. Three independent experiments were performed for each treatment condition. Root samples from all cuttings were cut and immediately stored at -80°C until required for RNA extraction. Total RNA from the root samples was extracted using TRIzol reagent (Invitrogen, Carlsbad, CA, United States). The quality and integrity of the RNA samples was confirmed and assessed, no sign of degradation was observed. cDNA library construction and Illumina sequencing were performed according to the manufacturer’s instructions (Illumina, San Diego, CA, United States). RNA sequencing (RNA-seq) was performed by the Beijing Genomic Institute (BGI, Shenzhen, China).

The *de novo* assembly of RNA-seq in the absence of a reference genome was accomplished using Trinity (Grabherr et al., 2011). Trinity combines the reads with a certain length of overlap to form longer fragments, known as contigs. These contigs were subjected to sequence clustering to form longer sequences. Such sequences were defined as unigenes (Zhou et al., 2017). For unigenes annotation, seven public databases were used, including the NCBI non-redundant protein sequence (NR), Swiss-Prot protein, euKaryotic Ortholog Groups (KOG), NCBI nucleotide sequence (NT), Kyoto Encyclopedia of Genes and Genomes (KEGG), protein families (Pfam), Gene Ontology (GO), and Intersection databases. The RNA-seq data were deposited in the NCBI Gene Expression Omnibus (GEO) with accession number GSE138551.

### Analysis of Differentially Expressed Genes

Transcriptome data processing and differentially expressed gene (DEG) analysis were performed as previously described (Zhou et al., 2017). The expression levels of the unigenes were calculated using the Reads per kilobase per Million reads (RPKM) method (Mortazavi et al., 2008). DEGs were screened with a false discovery rate (FDR) threshold of 0.05 or less and an absolute log2 ratio of 1 or more. All the DEGs were mapped to each term of the GO databases, and significant pathways were defined based on a corrected *P*-value ≤ 0.05.

### qPCR Expression Analysis

Expression levels of five sodium/hydrogen antiporter (NHX) genes (Unigene43275, CL3276, CL2217, CL7345, and CL9910) were investigated by qPCR. The *SlActin* (CL19537) was used as the internal reference gene. The relative gene expression levels were quantified using the delta-delta-Ct method. The qPCR primers used are listed in Supplementary Table S1.

### Statistical Analyses

All experiments performed at least three biological and at least three technical repeats. Statistically significant differences were calculated based on the Student’s *t*-test, with *P* < 0.05 (^∗^) using one-way Analyses of Variance in SPSS (SPSS Inc., Chicago, IL, United States).

## Results

### Comparison Between Sexes in Leaf Physiological Traits

**FIGURE 1:**
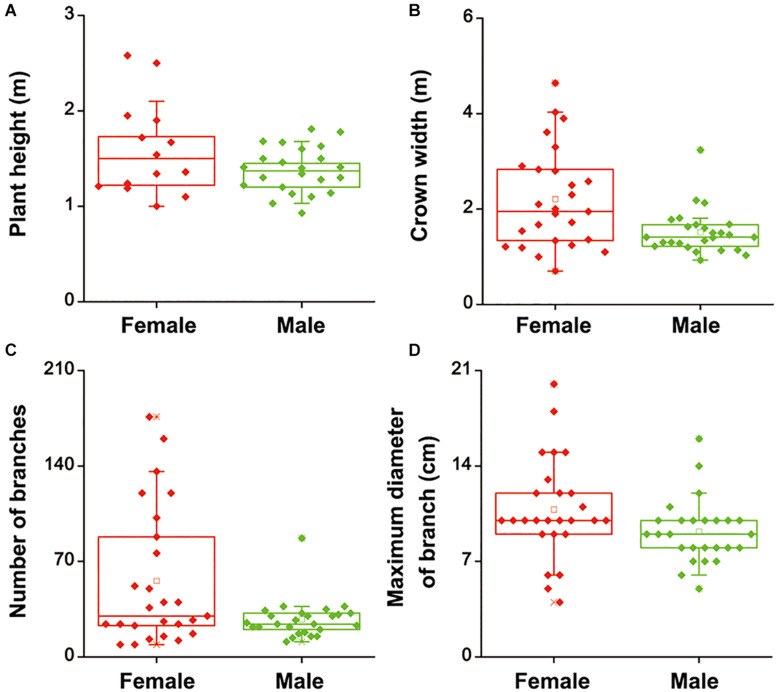
Height **(A)**, crown width **(B)**, number of branches **(C)**, and maximum diameter of branches **(D)** of female and male plants of *S. linearistipularis* from three natural populations distributed in saline-alkali soil. In the box plots, the hollow square () show the medians and center lines show the mean value; box limits indicate the 25th and 75th percentiles as determined by R software; whiskers extend 1.5 times the interquartile range from the 25th and 75th percentiles, and individual data points are represented by solid square.

**FIGURE 2:**
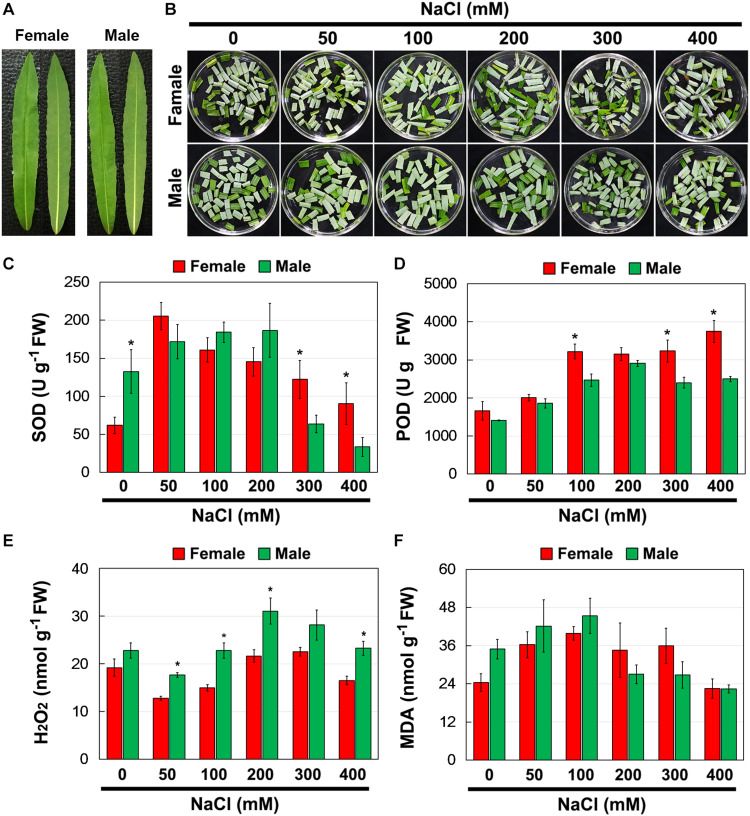
Phenotype **(A,B)**, SOD **(C)**, and POD **(D)** activities, and contents of H_2_O_2_ **(E)** and MDA **(F)** in leaves of female and male plants of *S. linearistipularis* under control and salinity stress (50 to 400 mM NaCl). Error bars represent *SE* (*n* = 5). Asterisks indicate significant differences between female and male plants (^∗^*P* < 0.05; Student’s *t*-test). Fw, fresh weight.

To investigate whether there were differences in salinity tolerance between female and male of *S. linearistipularis* plants, growth phenotypes from five populations in saline-alkali habitats were first investigated and compared. The average plant height, crown width, branch number, and maximum branch diameter of female plants were higher than those of male plants (Figures 1A–D). There were no significant morphological differences in female and male leaves (Figure 2A). The physiological traits of female and male leaves exposed to salinity (NaCl) stress were measured. Under the control conditions (0 mM), the male leaves showed significantly higher SOD activity than the female leaves (Figure 2B). After 50 to 200 mM NaCl treatment, there were no significant differences between sexes. At higher NaCl concentrations (300 and 400 mM), female leaves exhibited significantly higher SOD activity than males (Figure 2C). Under salinity stress, female leaves exhibited generally higher POD activity and lower H_2_O_2_ content than male leaves. However, there were no significant differences between female and male leaves under the control (0 mM) condition (Figures 2D,E). In addition, there were no significant differences in leaf MDA contents between the females and males under both the control and salinity stress (Figure 2F).

**FIGURE 3:**
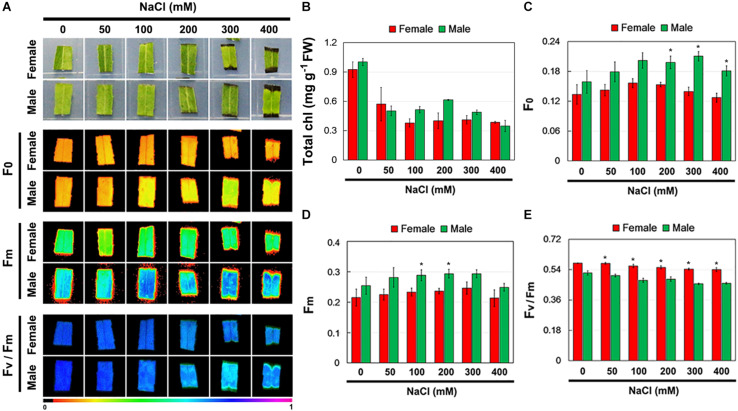
Chlorophyll (Chl) contents and Chl fluorescence parameters in leaves of female and male plants of *S. linearistipularis* under control and salinity stress (50 to 400 mM NaCl). **(A)** Minimum Chl fluorescence (F_0_), maximal Chl fluorescence (Fm), and maximal photochemical efficiency (Fv/Fm) images. The pseudocolored bar depicted at the bottom of the panel ranges from 0 (black) to 1.0 (purple). Chl content **(B)** and quantification of Chl fluorescence parameters *F*_0_ **(C)**, *F*_*m*_ **(D)** and *F*_*v*_/*F*_*m*_ **(E)**. Error bars represent *SE* (*n* = 6). Asterisks indicate significant differences between female and male plants (^∗^*P* < 0.05; Student’s *t*-test). Fw, fresh weight.

Further, the total Chl content and Chl fluorescence parameters of female and male leaves exposed to salinity stress were measured (Figure 3A). Salinity stress decreased total Chl content in both female and male leaves, but there were no significant differences between them under the control and salinity stress treatments (Figure 3B). Under the control conditions, there was no significant difference in *F*_0_, *F*_*m*_, and *F*_*v*_/*F*_*m*_ between the female and male leaves. However, under salinity stress, the *F*_0_ and *F*_*m*_ in male leaves were higher than those in female leaves, while the *F*_*v*_/*F*_*m*_ in male leaves was significantly lower than that in female leaves (Figures 3C–E).

### Comparison Between Sexes in Leaf Na^+^ Accumulation and Root Na^+^ Efflux

**FIGURE 4:**
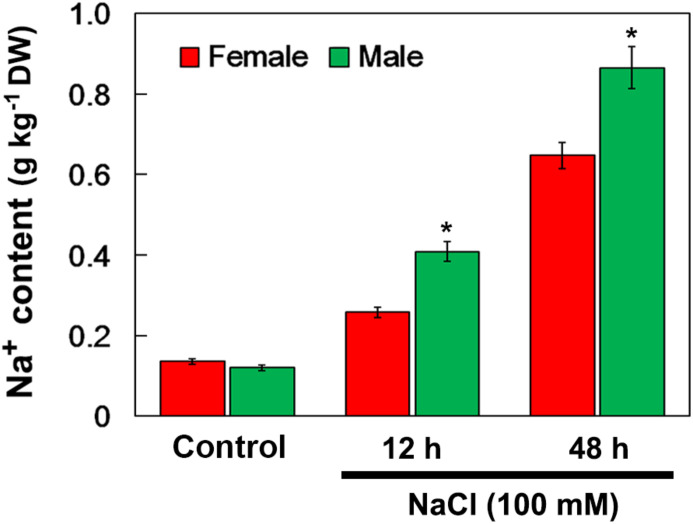
Na^+^ contents in leaves of female and male plants of *S. linearistipularis* under control and salinity stress (irrigation with 100 mM NaCl). Leaves were sampled 0 (control), 12 and 48 h after the initiation of salinity treatment. Error bars represent *SE* (*n* = 3). Asterisks indicate significant differences between female and male plants (^∗^*P* < 0.05; Student’s *t*-test). DW, dry weight.

When salinity (100 mM NaCl) was applied into the soil, the Na^+^ accumulation in the female and male leaves increased. After 12 and 48 h of NaCl treatment, the Na^+^ accumulation in male leaves was significantly higher than that in female leaves. However, there were no significant differences in Na^+^ accumulation between the female and male leaves under the untreated control conditions (Figure 4).

**FIGURE 5:**
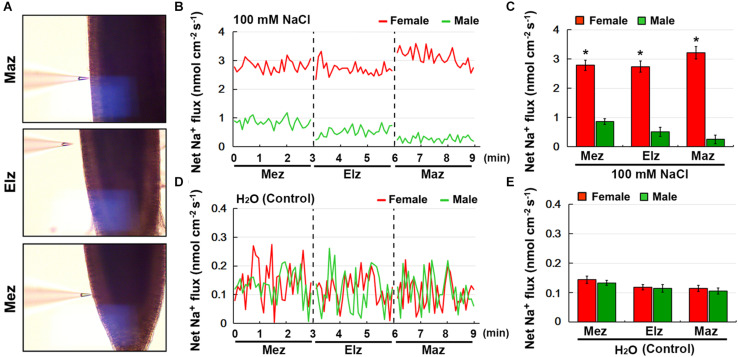
Net fluxes of Na^+^ in roots of female and male plants of *S. linearistipularis* under control and salinity stress (100 mM NaCl for 12 h). **(A)** The root zones measured by NMT technology. Mez, Meristem zone; Elz, elongation zone; Maz, mature zone. **(B)** Net Na^+^ fluxes in roots of female and male plants pretreated with 12 h incubation in 100 mM NaCl. Continuous flux was recorded for 9 min, 3 min per root zone. **(D)** The normal test solution (0.1 mM NaCl) was used as the control. **(C,E)** Mean Na^+^ fluxes from the roots of female and male plants under the condition of NaCl (100 mM) treatment and control (0.1 mM NaCl). Error bars indicate *SE* (*n* = 6). Asterisks indicate significant differences between female and male plants (^∗^*P* < 0.05; Student’s *t*-test).

Using the NMT technique, the net Na^+^ flux in female and male roots under salinity stress was monitored (Figure 5A). Transfer of NaCl-treated roots to Na^+^-free solution caused a significant Na^+^ efflux in both the female and male roots, including the root meristem zone (Mez), elongation zone (Elz), and mature zone (Maz) (Figure 5B). The rate of net Na^+^ efflux in female roots was significantly higher than that in male roots (Figure 5C). Under the control treatment, the rate of net Na^+^ efflux in female and male roots was not significantly different (Figures 5D,E).

### Comparison Between Sexes in Transcriptional Profiling in Roots

To investigate potential sexual differences in root Na^+^ flux, transcriptional sequencing of female and male roots under control (CK) and salinity stress was performed. In total, 220,256 transcripts (85.56% of all transcripts) in the control and NaCl-treated roots of both female and male plants were annotated in the following public databases, including the NR, NT, Swiss-Prot, KEGG, KOG, Pfam, and GO (Table 1).

**TABLE 1:** Number of functional annotations for all of the transcripts in public databases.

**Annotated Database**	**NR**	**NT**	**SwissProt**	**KEGG**	**KOG**	**Pfam**	**GO**	**Overall**
Number of transcripts	206,706	166,419	152,229	160,838	164,661	156,833	152,635	220,256
Percentage (%)	80.30%	64.65%	59.14%	62.48%	63.97%	60.92%	59.29%	85.56%

**FIGURE 6:**
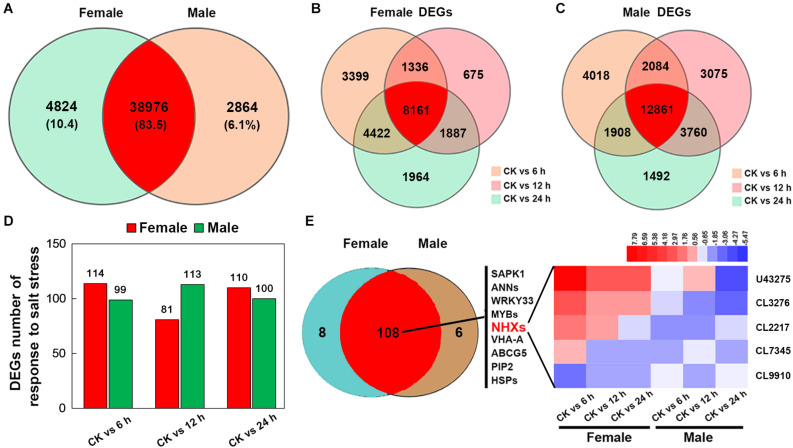
RNA-seq and analysis of salt-responsive genes in the roots of female and male plants of *S. linearistipularis*. **(A)** Venn diagrams of the total mapped genes expressed in the roots of female and male plants under control and salinity stress (100 mM NaCl). **(B)** Venn diagrams of DEGs identified in the roots of female and male plants under salinity stress for 6, 12, and 24 h. We used a false discovery rate ≤ 0.01 and the absolute value of log2Ratio ≥ 1 as the threshold to judge the significance of the differences in gene expression. **(C)** Number of DEGs in response to salt stress in the roots of female and male plants. **(D)** Venn diagrams of genes responding to salt stress in the roots of male and female plants and expression profile of key salt-responsive genes (sodium/hydrogen antiporter, NHX). Red rectangles represent the up-regulation of genes, while blue rectangles represent down-regulation. CK, control.

**FIGURE 7:**
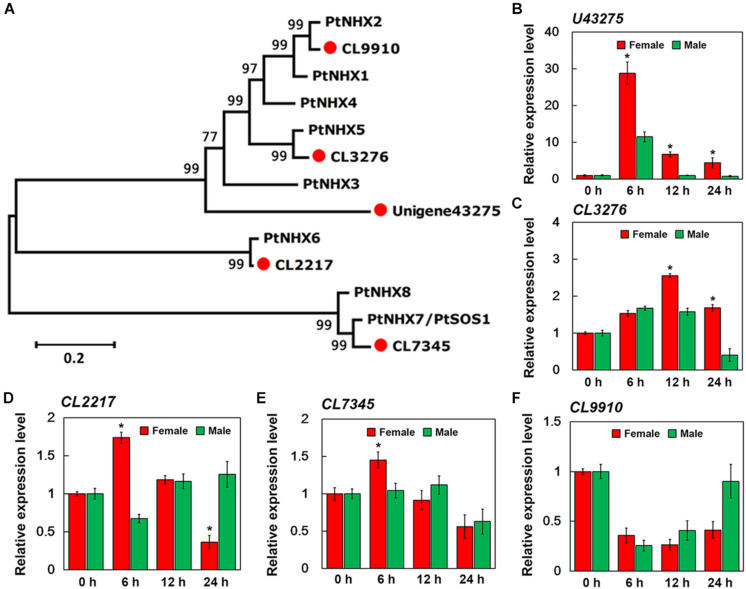
qPCR verification of expression of five sodium hydrogen antiporter (*NHX*) genes in the roots of female and male plants of *S. linearistipularis*. **(A)** Phylogenetic trees of *S. linearistipularis* NHX proteins with *P. trichocarpa* NHX family. **(B–F)** Expression of five *NHX* genes in female and male roots under salinity stress for 6, 12, and 24 h. Error bars indicate *SE* (*n* = 3). Asterisks indicate significant differences between female and male plants (^∗^*P* < 0.05; Student’s *t*-test).

After merging transcripts with the same annotation, a total of 46,664 unigenes were obtained. Out of all the genes, 4,824 were identified in female plants, and 2,864 were identified in male plants, and 38,976 (83.5% of all unigenes) were common to both the sexes (Figure 6A). Principal component analysis based on gene expression showed that the sequenced samples clustered well (Supplementary Figure S1). Using the RPKM method, the DEGs in female and male roots were screened. After 6, 12, and 24 h of NaCl treatment, 8,161 and 12,861 common DEGs were identified in female and male roots, respectively (Figure 6B). The GO enrichment analysis for DEGs showed that the response to the salt stress pathway was significantly enriched in both female and male plants (Supplementary Figure S2). At different time points of NaCl treatment, the number of DEGs involved in response to salt stress pathway was different between female and male plants (Figure 6C and Table 2). Furthermore, the number and expression levels of DEGs involved in reactive oxygen species pathways were different between the female and male plants (Table 2 and Supplementary Table S2). However, most of the DEGs involved in response to salt stress pathway were the same in both sexes; 108 DEGs are common to both sexes, 8 DEGs are specific to female plants, and 6 DEGs are specific to male plants (Figure 6D). The expression patterns of the common DEGs were different between the females and males under salinity stress (Supplementary Figure S3 and Supplementary Table S3). Notably, 5 *NHX* genes (U43275, CL3276, CL2217, CL7345, CL9910) were identified among the common DEGs, four of which (U43275, CL3276, CL2217, CL7345) exhibited higher expression levels in female roots than in male roots under salinity stress (Figure 6D). Sequence alignment showed that the five *NHX* genes were highly homologous to *PtNHX2*, *PtNHX3*, *PtNHX5*, *PtNHX6*, and *PtNHX7/PtSOS1* of *P. trichocarpa* (Figure 7A). The qPCR results were generally in consistent with the RNA-seq data (Figures 7B–F).

**TABLE 2:** Gene ontology (GO) enrichment analysis for the salt-responsive DEGs between females and males.

**Go term**	**Description**	**DEGs number**						**Rich ratio**					
		**Female**			**Male**			**Female**			**Male**		
		**6 h/ck**	**12 h/ck**	**24 h/ck**	**6 h/ck**	**12 h/ck**	**24 h/ck**	**6 h/ck**	**12 h/ck**	**24 h/ck**	**6 h/ck**	**12 h/ck**	**24 h/ck**
GO:0009651	Response to salt stress	252	162	211	229	251	248	0.59	0.38	0.49	0.54	0.59	0.58
GO:0009414	Response to water deprivation	200	130	155	181	172	200	0.71	0.46	0.55	0.64	0.61	0.71
GO:0006970	Response to osmotic stress	25	8	16	28	36	41	0.33	0.10	0.21	0.37	0.48	0.55
GO:0006979	Response to oxidative stress	539	317	440	435	517	485	0.61	0.35	0.49	0.49	0.58	0.54
GO:0000302	Response to reactive oxygen species	126	59	113	125	156	142	0.61	0.28	0.55	0.61	0.76	0.69
GO:0009737	Response to abscisic acid	87	39	67	61	101	95	0.51	0.23	0.39	0.36	0.59	0.56
GO:0009415	Response to water	39	13	22	26	32	24	0.76	0.25	0.43	0.50	0.62	0.47
GO:1901001	Negative regulation of response to salt stress	8	4	6	11	7	9	0.5	0.25	0.37	0.68	0.43	0.56
GO:1901002	Positive regulation of response to salt stress	12	8	12	12	10	7	0.70	0.47	0.70	0.70	0.58	0.41
GO:1902074	Response to salt	5	6	9	7	9	8	0.45	0.54	0.81	0.63	0.81	0.72
GO:0071472	Cellular response to salt stress	19	6	19	18	26	21	0.47	0.15	0.47	0.45	0.65	0.52
GO:0042631	Cellular response to water deprivation	8	1	1	7	3	6	1	0.12	0.12	0.87	0.37	0.75

## Discussion

### Sexual Difference in Physiological Response to Salinity Stress

Salinity stress can cause ion toxicity and osmotic imbalances, leading to secondary oxidative stress in plants (Chaves et al., 2009). Thus, salt exclusion or reducing salt uptake, preventing oxidative damage are the key defense mechanisms for plant adaptation to salinity (Chaves et al., 2009). In our study, salinity treatment increased the H_2_O_2_ content and activated the activities of antioxidant enzymes, SOD and POD, in female and male leaves. However, the female leaves showed higher SOD and POD activity and lower H_2_O_2_ content than the male leaves under salinity stress (Figure 2). The results showed that the antioxidant response of female and male leaves was different, and the antioxidant capacity of female leaves was stronger than that of male leaves under salinity stress. Stress can cause the decrease in *F*_*v*_/*F*_*m*_, which can reflect the degree of PSII damage, and then be used as an indicator of the ability of plants to tolerate environmental stress (Maxwell and Johnson, 2000). Under salinity stress, *F*_*v*_/*F*_*m*_ in female leaves decreased more slowly than that in male leaves (Figure 3E). This result suggests that the damage of salinity to PSII in female leaves was weaker than that in male leaves.

### Sexual Difference in Na^+^ Accumulation

Long-term exposure of plants to salinity stress results in Na^+^ accumulation in leaves, leading to toxic symptoms (Munns and Tester, 2008; Rahnama et al., 2010). The Na^+^ content in both female and male leaves significantly increased when exposed to salinity stress (Figure 4). However, under the untreated control conditions, there was no difference in Na^+^ content between female and male leaves. The Na^+^ accumulation in leaves is closely related to the Na^+^ uptake rates by roots and the rate of Na^+^ transport to shoots (Shi et al., 2002). NMT monitoring showed that the female roots had higher Na^+^ efflux rate than that of the male roots under salinity stress (Figures 5B,C). These results showed that the Na^+^ accumulation in leaves and Na^+^ efflux rate in roots were different in female and male plants, and less Na^+^ accumulation in female leaves than male leaves may be related to higher Na^+^ efflux rate in its roots under salinity stress.

### Sexual Difference in Transcriptional Response to Salinity Stress

Transcriptional analysis showed that salinity induced the differential expression of a large number of genes in female and male roots (Figure 6B). At different time points after salt treatment, the number of salt response-related DEGs in female and male plants was different (Figure 6C). However, most of these salt response-related DEGs are common to both sexes, including *SAPK1*, *WRKY33*, *VHA-A*, *ABCG5*, and *PIP2* genes, and many members from the *ANN*, *MYB*, *NHX*, and *HSP* gene families (Figure 6D). These genes are reported to be involved in plant salt stress responses (Jiang and Deyholos, 2009; Matsuda et al., 2014; Zhu et al., 2015; Lv et al., 2017; Lou et al., 2018; Li et al., 2019). Under salt stress, Na^+^ transport was directly regulated by the NHX family at endomembranes (Bassil and Blumwald, 2014). Genome analysis of multiple species has shown that the NHX family consists of about 5 to 9 members (Bassil and Blumwald, 2014). In *P. trichocarpa*, the NHX family consists of 8 members (Tian et al., 2017). Five *NHX* genes were identified from the transcriptome of *S. linearistipularis* roots by sequence alignment, and all of them appeared in common salt response-related DEGs of both sexes (Figure 6D). By phylogenetic analysis, the five *SlNHXs* were homologous to *PtNHX2*, *PtNHX3*, *PtNHX5*, *PtNHX6*, and *PtNHX7/PtSOS1*, respectively (Figure 7A). RNA-seq and qPCR experiments showed that the salt-induced expression of four *NHX* genes in female roots was higher than that in male roots (Figures 7B–F). Thus, we speculated that the sexual difference of Na^+^ efflux between female and male roots may be closely related to the differential expression of *NHXs* in both sexes under salinity stress. These results suggest that the salinity tolerance mechanisms in female and male plants are the same, but the sexual differences in salinity tolerance between them may be caused by the differential expression of these salt-responsive DEGs, such as *NHXs*. However, the reasons for the differential expression of these salt-responsive DEGs in both sexes are unclear and need to be further investigated. Of course, we cannot completely rule out the regulatory roles of female and male specific DEGs in the sexual difference of salinity tolerance.

In *P. cathayana*, previous studies have shown that salinity tolerance in females was weaker than that in males (Chen F. et al., 2010). The result is different from our observation, suggesting that the salinity tolerance mechanism of females and males in *P. cathayana* and *S. linearistipularis* is different. This difference may be related to species characteristics and habitats. The leaf morphology of *P. cathayana* was significantly different between females and males, but there was no difference in the leaf morphology between females and males in *S. linearistipularis*. In addition, the habitats of *P. cathayana* and *S. linearistipularis* are different. Saline-alkali habitat may have led to the evolution of a salinity tolerance mechanism in *S. linearistipularis*, which is different from that in *P. cathayana*. This observation needs to be verified in more species from the genus *Salix*. Recent studies have shown that *S. paraplesia* females are more drought tolerant and better self-protective ability than males from high altitude (Liao et al., 2019).

## Conclusion

We identified differences in salinity tolerance between female and male plants of *S. linearistipularis* by comparing the physiological traits and transcriptional profiling. In female roots, the differential expression of salt-response genes, especially *NHX*s, may cause stronger Na^+^ efflux, which further leads to less Na^+^ accumulation in leaves than in male roots. Lower Na^+^ accumulation in female leaves may be associated with higher antioxidant enzyme activity and lower H_2_O_2_ levels. It is inferred from the above results that female plants have stronger salinity tolerance than male plants. Therefore, in the afforestation process of *S. linearistipularis* for greening and ecological restoration of saline-alkali soil in the future, different salinity tolerance characteristics of female and male plants should be considered.

## Data Availability Statement

The datasets generated for this study can be found in the NCBI Gene Expression Omnibus (GEO), GSE138551.

## Author Contributions

SF, AZ, YB, and SL designed the experiments. SF, AZ, HS, HM, and XZ performed the experiments. SF, SM, KQ, and YB analyzed the data. SF and AZ wrote the manuscript. All authors contributed to the article and approved the submitted version.

## Conflict of Interest

The authors declare that the research was conducted in the absence of any commercial or financial relationships that could be construed as a potential conflict of interest.

## Supplementary Material

Supplementary Figure 1 Principal component analysis (PCA) based on gene expression from 24 sequenced samples.

Supplementary Figure 2 Scatterplot of GO pathways enriched for differentially expressed genes (DEGs) in the roots of female and male plants of *S. linearistipularis*. The rich factor is the ratio of the number of annotated DEGs in a given pathway term to the number of all genes annotated in the pathway term. The *Q* value is the corrected *P*-value and ranges from 0 to 1. The size of the circles indicates the number of genes. The top 20 enriched pathway terms in the KEGG database are listed. The red arrow indicates the significantly enriched response to the salt stress pathway.

Supplementary Figure 3 Expression profiles of 108 salt-responsive DEGs in female and male roots under salt stress. The bar represents the scale of the expression levels of each DEGs (log_2_ RPKM) in the heat map. The red rectangles represent the up-regulation of DEGs, and blue rectangles represent down-regulation. All information for each DEGs can be found in Supplementary Table S3.
