# Supplementary figures and images for: Sexual Differences in Physiological and Transcriptional Responses to Salinity Stress of Salix linearistipularis
Source: Front Plant Sci. 2020 Oct 19;11:517962. doi: 10.3389/fpls.2020.517962 (PMC7604296; doi:10.3389/fpls.2020.517962)

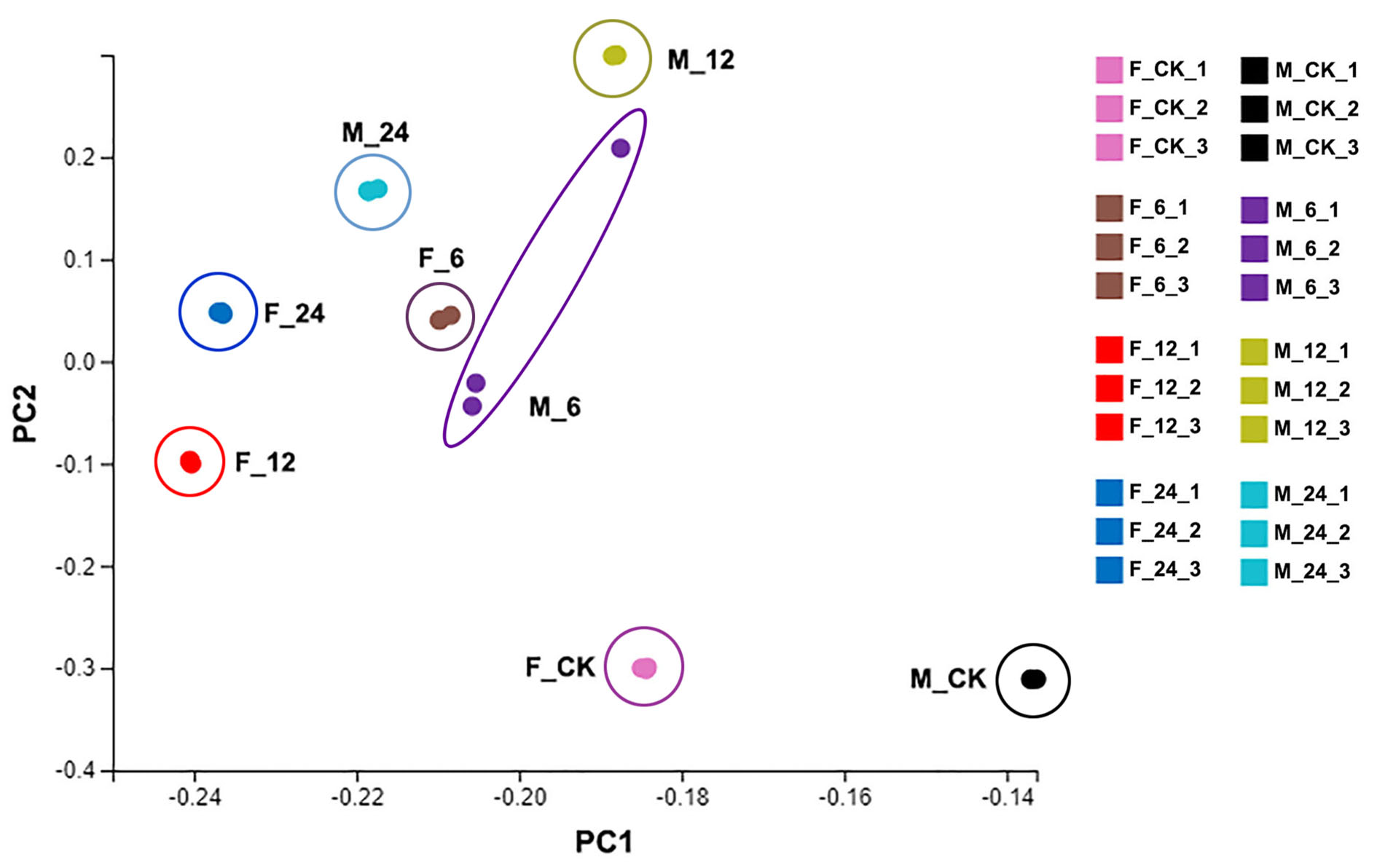

Supplement: Supplementary Figure 1 — Principal component analysis (PCA) based on gene expression from 24 sequenced samples. [file Image_1.TIF]

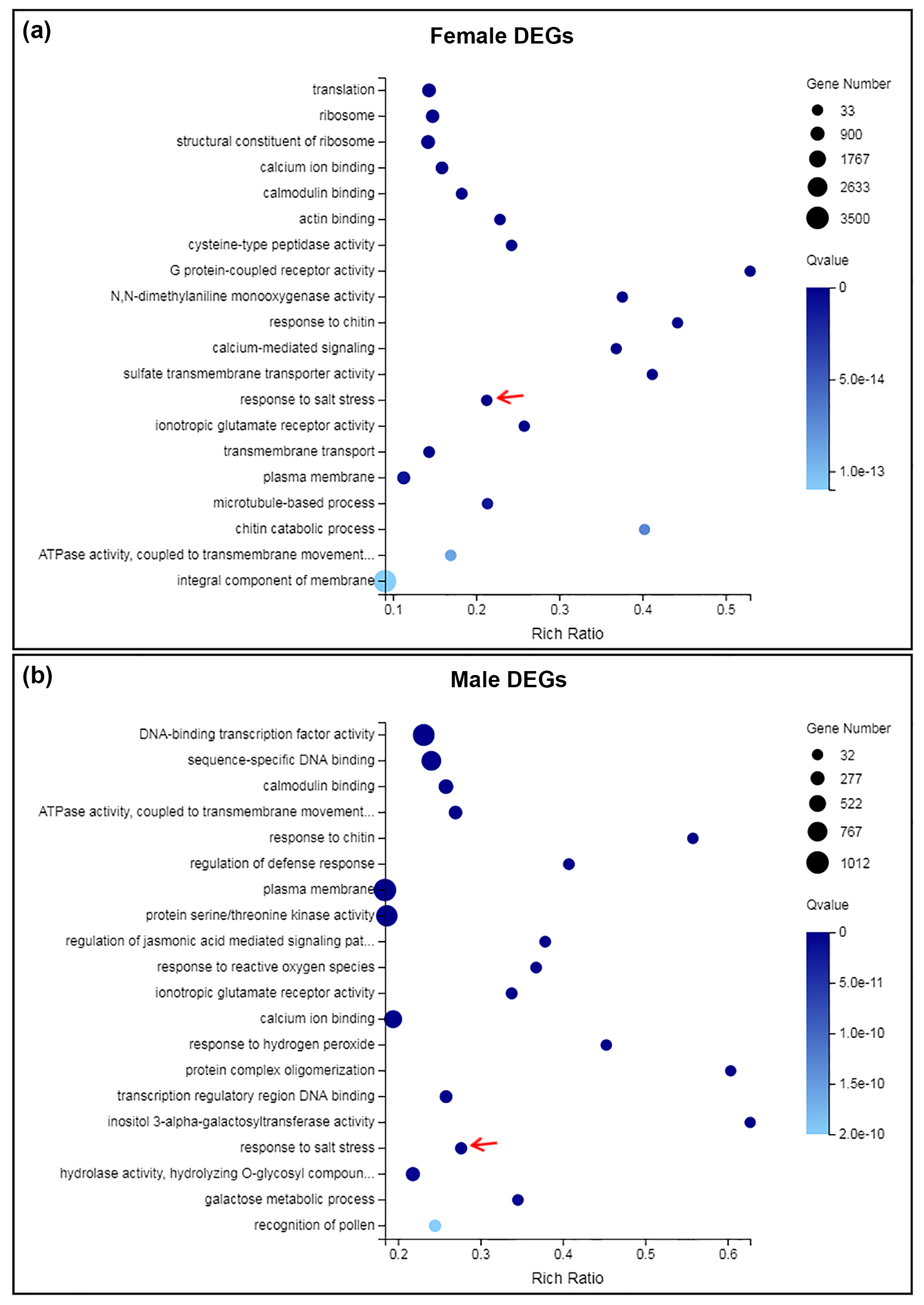

Supplement: Supplementary Figure 2 — Scatterplot of GO pathways enriched for differentially expressed genes (DEGs) in the roots of female and male plants of S. linearistipularis. The rich factor is the ratio of the number of annotated DEGs in a given pathway term to the number of all genes annotated in the pathway term. The Q value is the corrected P-value and ranges from 0 to 1. The size of the circles indicates the number of genes. The top 20 enriched pathway terms in the KEGG database are listed. The red arrow indicates the significantly enriched response to the salt stress pathway. [file Image_2.TIF]

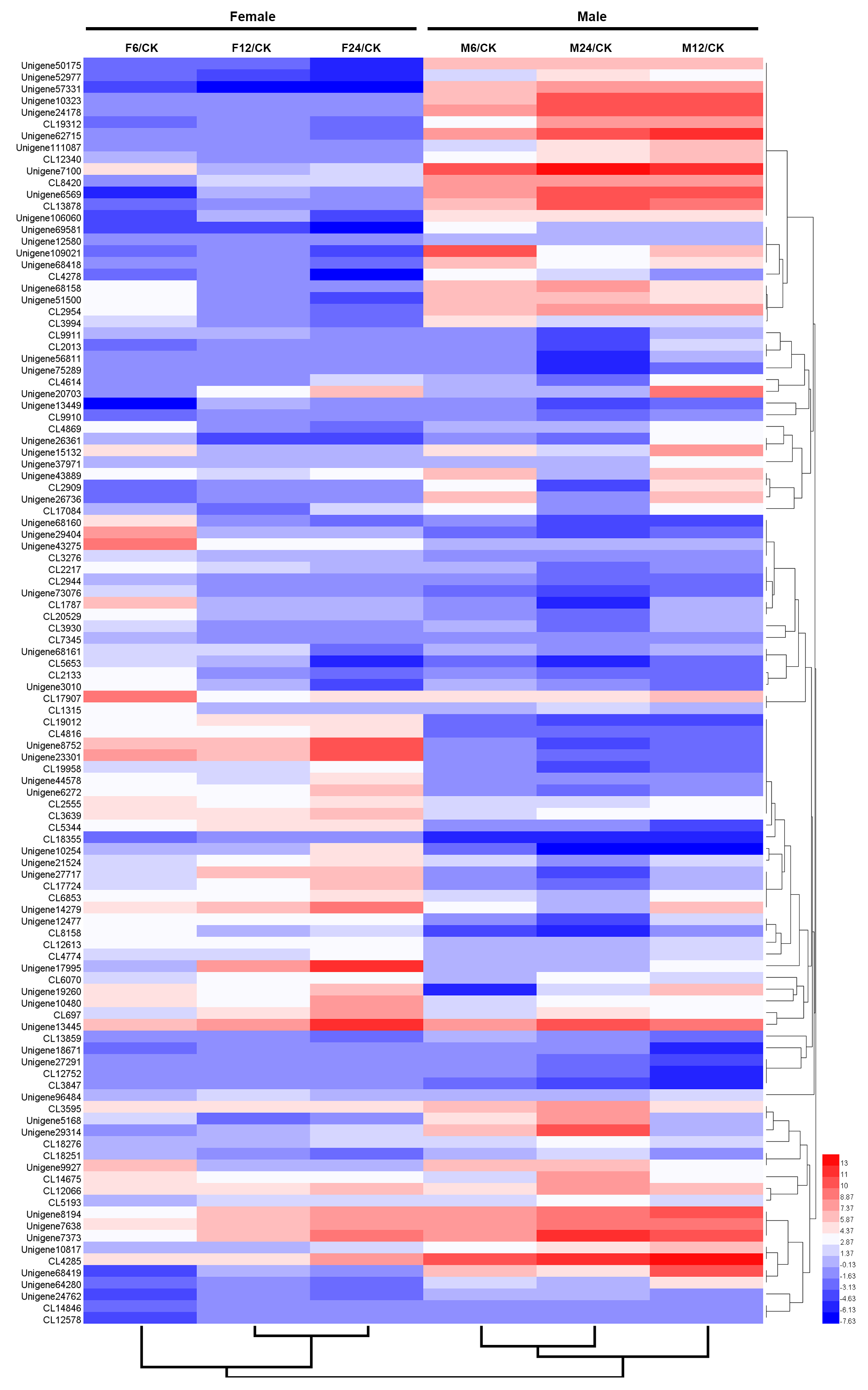

Supplement: Supplementary Figure 3 — Expression profiles of 108 salt-responsive DEGs in female and male roots under salt stress. The bar represents the scale of the expression levels of each DEGs (log2 RPKM) in the heat map. The red rectangles represent the up-regulation of DEGs, and blue rectangles represent down-regulation. All information for each DEGs can be found in Supplementary Table S3. [file Image_3.TIF]
